# BUS TRIPS—A Self-Management Program for People with Cognitive Impairments after Stroke

**DOI:** 10.3390/ijerph14111353

**Published:** 2017-11-07

**Authors:** Emma Carlstedt, Susanne Iwarsson, Agneta Ståhl, Hélène Pessah-Rasmussen, Eva Månsson Lexell

**Affiliations:** 1 Department of Health Sciences, Lund University, P.O. Box 157, SE-22100 Lund, Sweden; Susanne.Iwarsson@med.lu.se (S.I.); Eva.Mansson_Lexell@med.lu.se (E.M.L.); 2 Department of Technology and Society, Lund University, P.O. Box 118, SE-22100 Lund, Sweden; Agneta.Stahl@tft.lth.se; 3 Department of Neurology and Rehabilitation Medicine, Skåne University Hospital, SE-22185 Lund-Malmö, Sweden; Helene.Pessah@skane.se; 4 Department of Clinical Sciences, Lund University, P.O. Box 117, SE-22100 Lund, Sweden

**Keywords:** bus travelling, feasibility, participation, public transport, self-efficacy, stroke

## Abstract

Stroke is a major cause of disability worldwide and different types of impairments can affect the individual’s ability to manage everyday activities such as travel that is essential for participation in society. The purpose of this study was to investigate the feasibility of a new self-management intervention (BUS TRIPS) focusing on travelling by bus, and potential contributions to an improved ability to travel by bus for people with cognitive impairments after stroke. This is a pilot study of five individuals, utilizing a multiple case study design with a mixed methods approach. Assessments (Stroke Impact Scale, General Self-Efficacy Scale and Life Satisfaction Scale-11, Item 1) were performed before, two weeks after, and three months after the program. The data collection also comprised reflection notes from the group leaders (an occupational therapist and a physiotherapist), a semi-structured group interview and an individual phone survey. The feasibility of the intervention was presented in four sub-categories: (1) appreciated group format despite too short sessions; (2) the importance of skilled leaders and motivated participants; (3) session material adequate but needs minor revision to fit the target group; and (4) homework is valuable but reflective group discussions must be supported. The narratives of each case showed that all participants made some progress related to travelling by bus, but the overall positive results could not be verified by all of the quantitative assessments. We conclude that the BUS TRIPS intervention is feasible and can potentially contribute to an improved ability to travel by bus for the target group. Future studies is called for, and should focus on recruitment challenges, to clarify assessments that would be suitable to use in larger scale clinical trials and during subsequent implementation in clinical practice.

## 1. Introduction

Stroke is a major cause of disability worldwide, causing different types of impairments that can affect the individual’s ability to manage everyday activities and reintegration in the community [1,2]. Barriers in the outdoor environment are related to avoidance of leaving home [3] and outdoor mobility, including ability to travel, is essential for participation in society [4,5]. Because many people with stroke cease to drive a car [6], they often have to rely on family and friends or use public transport to move around in society. Although many people with stroke want and need to travel with public transport, only a minority have the ability [7,8], mainly caused by an inaccessible travel chain (e.g., planning the trip, walking to the bus-stop, buying tickets, and getting on and off the bus) [9,10]. Difficulties using public transport among people with stroke appear for example because their cognitive impairments (CIs) make it difficult to handle different tasks such as manage reservations, buying tickets, and handling orientation in a diversity of challenging environments [7,9,11]. Loss of confidence is another aspect common among people with stroke [12], which is associated with a reduced use of public transport [13,14].

There are very few studies addressing rehabilitation interventions focusing on public transport and outdoor mobility, for people with stroke. Logan et al. [12] showed how people with stroke who participated in a rehabilitation intervention focusing on outdoor mobility were significantly more likely to travel, compared with a control group. Still, those in the intervention group were not more satisfied with their outdoor mobility. The authors concluded that to improve the outcome of similar studies, participants’ confidence should be acknowledged. Barnsley et al. [15] added that also people with CIs should be included in such interventions.

One way to address, e.g., aspects of confidence, is to use self-management strategies (SMs) when delivering the intervention. SMs make participants more active and involved throughout the intervention, with the intention to increase their self-efficacy at the core. Typically, this type of method is used in health care to support people with disabilities to learn different skills to be applied in everyday life activities [16]. SM interventions have been used internationally, but only recently for people with stroke, especially for those with CIs [17]. Many stroke specific SM programs focus on the nature and consequences of a stroke as well as lifestyle factors for reducing the risk of a new stroke [18,19]. Warner et al. [20] concluded that some stroke SM programs indicate positive effects in terms of enhanced participation. A recent study showed positive effects on self-efficacy in participation in everyday life activities for people with mild–moderate stroke [21], even though it did not focus on public transport. Thus, it is evident that there is a need to develop and test interventions that utilize SM skills to enhance participation [22] among people with cognitive impairments after stroke [17] focusing on public transport.

We developed a new program: BUS TRIPS (BUS Travel for Improved Participation in Stroke survivors) based on previous knowledge gained from SM interventions targeting people with CIs in Multiple Sclerosis [23,24] and rehabilitation interventions for people with acquired brain injury [25]. Commonly, rehabilitation interventions comprise numerous interacting mechanisms, and are therefore often complex [26]. Accordingly, the first step towards developing such a program is to investigate the feasibility in terms of its content, delivery, recruitment, and suitable outcome measures, prior to evaluating its effects [26]. The aim of this study was twofold: (1) to investigate the feasibility of the BUS TRIPS intervention and (2) to investigate potential contributions to an improved ability to travel by bus for people with cognitive impairments after stroke.

## 2. Materials and Methods

### 2.1. BUS TRIPS

When developing BUS TRIPS, we presented a draft of the program to people with stroke, their family members, rehabilitation professionals, and researchers and used their comments to refine the program prior to the study.

BUS TRIPS used in this study consisted of seven 2 h sessions (group and individual), performed once per week, led by an occupational therapist and a physiotherapist. During the first session, each participant identified self-perceived problems in relation to activities linked to travelling by bus that s/he wanted to participate in. All smaller parts, that is, tasks of activities in a travel chain perspective [10], were considered. During this process we used the Canadian Occupational Performance Measure (COPM) [27]. Based on the information gathered, participants set goals for themselves, that is, which activities they wanted to be able to participate in. All group sessions comprised short lectures, discussions, and skills practice. In between sessions, participants performed individual homework, reflecting the different topics discussed. For example, a lecture of the main topic—goal setting—started the second session, and was followed by a discussion among the participants, and later they had the opportunity to practice their goal setting skills. This was also focus for their homework, and in the following session they were able to share individual experiences in the group. Other sessions taught Problem Solving Theory, activity analysis where different activities were broken down into smaller parts: tasks. Compensatory techniques in relation to travelling as well as learning how to communicate aspects important for bus travels were also included in the program. During individual sessions, participants were able to practice travelling by bus in a real life context taking a standpoint in each participant’s goals. The individual sessions were always performed together with one of the intervention leaders. In between the individual sessions, participants had the opportunity to contact the group leaders, and in some cases the group leaders contacted participants to follow-up certain aspects in relation to their homework. An overview of the BUS TRIPS intervention is presented in Table 1.

### 2.2. Participants

Participants were recruited from a local part of the national quality register of stroke incidents in Sweden (Riks-Stroke), based on the following criteria: stroke onset 6–12 months ago, age ≥ 55 years, self-reported independent mobility indoors and outdoors at the three-month post stroke evaluation, agreement to participate in a screening of CIs, and an interest in travelling by local public transport. Fifty-nine potential participants were contacted by phone by the first author (EC), a registered nurse with experience in data collection with people with stroke, who posed questions about experienced difficulties when travelling with local buses. This resulted in 11 possible participants. However, five of those had no time to participate, leaving us with six persons who were screened for and/or stated self-perceived CIs. Since one of these withdrew before starting the program due to a lack of energy, five participants (two women, three men; mean age = 72.2 years) completed the program. The intervention was performed during spring, and at a location in the south of Sweden. All participants had access to local busses and lived in urban or sub-rural areas. For an overview of their profiles, see Table 2. The study was conducted in accordance with the Declaration of Helsinki, and the Regional Ethical Review Board in Lund, Sweden (Ref. No. 2012/174) approved the study. Prior to the intervention, all participants submitted written informed consent.

### 2.3. Study Design

This study has a multiple case study design [40] with a mixed methods approach [41], including both qualitative and quantitative data.

### 2.4. Data Collection

Data were collected through different sources: (1) demographics, (2) baseline data, (3) three quantitative assessments, measured before and two weeks after program completion, and at a three-month follow-up (Stroke Impact Scale, General Self-Efficacy Scale and Life Satisfaction Questionnaire, Item (1), (4–5) group leaders’ qualitative reflection notes (from group and individual sessions), (6) a qualitative semi-structured group interview (performed during last group session), and (7) an individual phone survey (performed one week after program completion). For an overview (see Table 3). All data collection, except from the reflection notes, was administered by EC; she was not involved in the program.

#### 2.4.1. Program Feasibility

To investigate the feasibility of BUS TRIPS, we used three data sources: the leaders’ reflection notes (from the group sessions), the semi-structured group interview, and the individual phone survey. The data from the leader’s reflection notes included, for example, attendance, time distribution, how the lecture material worked, and any issues/uncertainties arising during the sessions. The semi-structured group interview was performed in the end of the last group session, led by EC and an observer. This audiotaped interview lasted 80 min and was transcribed verbatim. The interview included questions about the program such as “How did you experience the homework?” Last, an individual phone survey was performed where each participant answered questions such as “Could the leaders present the course material in a way you understood?” Each question was answered on a seventh grade scale (1 = not at all and 7 = excellent). All data sources are presented in Table 3.

#### 2.4.2. Improved Ability to Travel by Bus Due to BUS TRIPS

To describe each participants’ possible improved ability to travel by bus, we used five data sources: demographics, baseline data, the three assessments, the leaders’ reflection notes (from the individual sessions), the semi-structured group interview, and the individual phone survey (Table 3). The baseline data were used to describe activity and participation, environmental barriers, cognitive impairments and depressive symptoms of the participants. The three assessments captured stroke impact on participation (SIS), general self-efficacy (GSE), and life satisfaction (Lisat-11, Item 1). The leaders’ reflection notes from the individual sessions followed the same structure as those from the group sessions. During the semi-structured group interview, questions were posed in relation to possible improved ability to travel by bus due to BUS TRIPS, e.g., “If, and in what way have the BUS TRIPS increased your ability to travel by bus?” The individual phone survey included questions on possible improved ability to travel by bus due to BUS TRIPS, e.g., “Overall, how much do you think this program have helped you to travel more by bus?”.

### 2.5. Data Analysis

The first author (Emma Carlstedt) performed all analysis in an interactive process involving all co-authors.

#### 2.5.1. Cross Case Analysis: Program Feasibility

A cross case analysis [40] was used to capture program feasibility. The qualitative data from the leader’s reflection notes (from the group sessions) and the semi-structured group interview were analyzed deductively through directed content analysis [42], focusing on program delivery and program content as the main categories. Reading the material repeatedly, text units related to each of the main categories were first compiled. The data within each of the main categories were then further analyzed into codes and sub-categories. To support the descriptions, quantitative data from the phone surveys were used to verify the qualitative data, but the results from the survey are also presented separately.

#### 2.5.2. Within Case Analysis: Improved Ability to Travel by Bus Due to BUS TRIPS

A within case analysis [40] was conducted for each participant, embedding the quantitative data in the qualitative data [41] to create rich narratives of each participant. First, all data were read repeatedly. Baseline data were summarized and used to describe each case profile. Further analyses aiming to describe goals, barriers, concerns, and possible solutions from each case’s process during and after the BUS TRIPS were performed. The analysis was first performed with qualitative data from the group interview and the leader’s reflection notes (from the individual sessions). Using an embedded design [41] allowed us to merge the quantitative phone survey data into the narratives, in order to support the qualitative results and to give a rich and comprehensive picture of each case. Last, the results from the three assessments were merged and presented at the end of the narratives.

## 3. Results

### 3.1. Cross Case Findings: Program Feasibility

Each of the two main categories (program delivery and program content) comprised two sub-categories (Table 4). In addition, the results from the phone survey are presented in Table 5.

#### 3.1.1. Program Delivery

##### Appreciated Group Format despite Sessions That Are Too Short

The group format was very valuable for the participants because it made it possible for them to meet and share experiences with others with the same difficulties. One participant said: “It is social and you can exchange thoughts and give each other tips” (Kent). Four of the participants were present at all sessions. The fifth could not attend the last group session. They wanted to have the sessions closer to home, but at the same time said the frequency (one session per week) was just right, “It is just what you can set aside time for” (Carl).

Both leaders were present all sessions except from one when one of the leaders fell ill. Both emphasized the need of being two to be able to cover the content as planned. Although the participants did not seem to mind, the leaders experienced that the time for the sessions was overall too short to catch all content as planned and at the same time make room for breaks. They argued that sessions should be extended to three hours but that individual sessions should be reduced to one, since only one had been used during the program. The occupational therapist leader said: “It is really difficult to make enough time. We probably need three hours for every session to make room for a break.” They also suggested changing orders between some sessions: “The communication session should be placed earlier in the schedule. It is a bit strange to place it after they have finished their individual tasks” (occupational therapist leader).

##### Importance of Skilled Leaders and Motivated Participants

Due to the phone survey, the participants felt the leaders had the right skills and explained the course material using simple language (Table 5). They appreciated that the leaders were skilled and calm, which made them feel secure during the individual sessions. “She is very calm and very thoughtful, so even though it was the first time I travelled, I did not feel stressed and worried. Instead I felt very calm because I felt secure with her” (Elisabeth).

The leaders expressed the importance of revising the recruitment process, as not all participants were motivated to travel by bus or expressed no problems when travelling by bus during the program, although all participants stated such interests prior to participation in the intervention. “For some participants it was not obvious that they needed to practice travelling by bus. They experienced they could travel in other ways which worked well” (Physiotherapist leader).

#### 3.1.2. Program Content 

##### Session Material Adequate but Needs Minor Revision to Fit the Target Group

Participants ranked the quality of the material as good to very good during the phone survey (Table 5). They thought they had received correct information in an understandable format, at an appropriate pace, and learnt a lot about cognitive impairments following stroke. They appreciated having learnt problem-solving skills such as dividing larger activities into manageable tasks. According to the leaders, most of the material was well suited to fit the participants’ needs. “It seemed like the participants assimilated the material, since they asked supplementary questions and shared own experiences” (physiotherapist leader).

Still, leaders claimed that the participants needed much support to understand some of the material. They also meant that, preferably, defining activity problems and goal setting should be performed individually prior to the program since this was difficult to pursue within the group during the program.

In turn, participants emphasized that some presentations were superfluous, and already well known: “The lecture of the travel planner and cell phone did not give much, because I knew it since before” (Elisabeth). Instead, they lacked information about, for example, whether they were allowed to bring an accompanying person for free. Such knowledge would make them feel more secure. They also suggested including practical use of mobility devices; they wanted to touch, try out, and evaluate them.

Both participants and leaders recommended an adaption of the program material to participants’ needs, for example, printing in larger font size suitable to also those with visual impairments. 

##### Homework Is Valuable but Reflective Group Discussions Must Be Supported

The participants appreciated the homework and ranked it highly at the phone survey (Table 5). It was positive and stimulating without being a burden. “It has been fun and instructive and I have felt well doing it” (Kent). The leaders described how introducing and monitoring the homework often went well, but suggested that the presentations also should be in writing to avoid misunderstandings. To improve the homework discussions, the leaders stated a need to develop strategies to involve all participants. The participants verified this need as they rated the leaders’ skills to initiate group discussions slightly lower than other skills. Still, they were pleased with the feedback they received (Table 5).

### 3.2. Within Case Findings: Improved Ability to Travel by Bus Due to BUS TRIPS

Elisabeth is living with her husband in a single-family house. She is a slow walker and uses a rollator (i.e., wheeled walker) for outdoor mobility. She does not drive a car but travels with her husband. Before the stroke, she went by bus on a daily basis. At baseline, Elisabeth expressed how the design of buildings and surroundings affected her participation. She complained of getting tired and losing concentration faster than she did pre-stroke. She also found it difficult to plan ahead and to stay attentive. Elisabeth reported several depressive symptoms (Table 2).

According to the physiotherapist leader’s reflection notes during the individual session with Elisabeth, her goal was to overcome the fear of travelling by bus, and to be able to travel independently. She identified problems with getting on and off the bus, and not getting a seat. She suggested alternative solutions, for example, avoiding travelling during rush hours and asking the driver to wait to drive off until she was seated. During the practice trip, Elisabeth managed to use a cane instead of a rollator. This had been an obstacle but after the successful ride, the physiotherapist leader considered Elisabeth to be secure enough to try it again with her husband and later on by herself.

Directly after the program Elisabeth felt more self-confident, less scared and uncertain: “The fear and the uncertainty has decreased now and it is quite important that you get some self-confidence” (Elisabeth). Due to the phone survey (Table 5), she stated that the program had facilitated her participation in activities outside home, although the number of times she moved around in the community did not increase. The opportunity to practice travel skills with a skilled leader encouraged her to travel more by bus: “Even though it was the first time I travelled by bus; I was not stressed or worried. Instead I felt very calm, because I felt secure with her (physiotherapist leader) and that was good” (Elisabeth). She also said the leaders taught her skills such as to divide larger activities into smaller tasks to identify problems in the travel chain. Solving problems at the task level also helped her to overcome obstacles in the activity as a whole without being overwhelmed.

**Figure 1:**
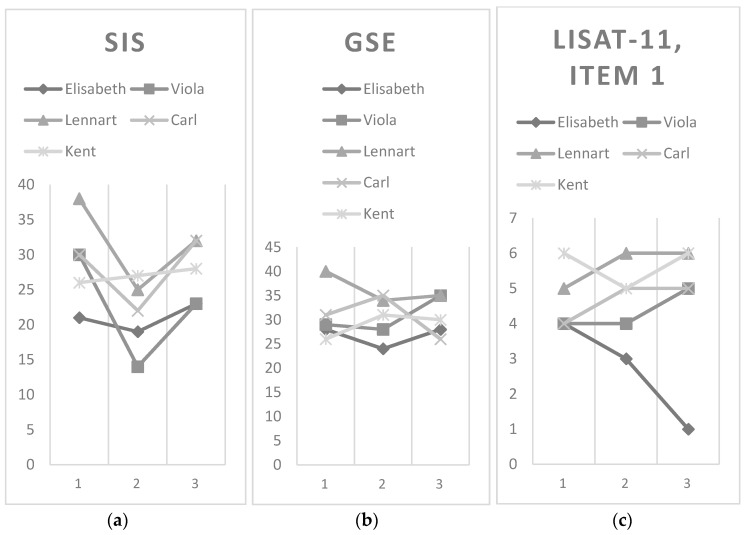
Assessment results from baseline to three months’ post BUS TRIPS, *N* = 5. (**a**) SIS (Stroke Impact Scale), perceived impact on activity and participation part. Score range: 1 = All the time to 5 = Never. (**b**) GSE (General Self-Efficacy scale): Score range: 1 = Not at all true to 4 = Exactly true (Higher scores = higher sense of GSE). (**c**) Lisat-11 (Life Satisfaction Questionnaire-11). Score range: 1 = Very unsatisfied to 6 = Very satisfied.

Elisabeth felt the stroke had affected her societal participation, particularly in leisure activities at all three data collections, according to SIS. She rated her general self-efficacy lower after, than before the program, but at the three-month follow-up, ratings were back to baseline. Regarding life satisfaction in general, she felt quite satisfied with life at baseline, but this decreased over time (Figure 1).

Viola is living with her husband in an apartment. She is a slow walker using a rollator outdoors. She has no access to a car. Pre-stroke she traveled by bus several times per week but expressed that access to transport and the environment as such affected her societal participation. She described difficulties related to CIs, for example, tiredness, loss of concentration, and difficulties with attention, expression, and remembering things. She reported several depressive symptoms (Table 2).

One of the problems Viola expressed when travelling by bus was that because both she and her husband used rollators, sometimes they were not able to travel together as the buses only accepted two rollators onboard. According to the physiotherapist’s reflection notes from the individual session with Viola, she needed support during goal setting and to find solutions to her travel difficulties. Still, during the practice trip Viola used different strategies to solve problems, for example asking other people for help. Although Viola was an experienced bus traveler already, she expressed that the program had helped her to become a more established bus traveler: “I don’t think much anymore, that I am taking the bus. For now, it has become habit” (Viola). She also said during the phone survey (Table 5) that the program had increased her overall participation in societal activities and that the leaders had encouraged her to try out new strategies. She gave examples of strategies (e.g., goal setting) that she had learnt and had started to use in everyday activities. “I will start with that, point out what to do each day and then do it goal-oriented. I have begun doing it more and more already” (Viola).

Viola perceived the stroke had affected her participation, which decreased from baseline to follow-up, according to SIS. Her general self-efficacy increased at follow-up as well as her life satisfaction in general (from quite satisfied at baseline and after program to satisfied at follow-up) (Figure 1).

Lennart is living with his wife in a single-family house. He does not use any mobility device but is a slow walker. He does not drive himself but travels in the car with his wife. He rarely traveled by bus. Lennart did not express any environmental barriers influencing participation. He reported some CIs such as getting tired easily, losing concentration, having attention deficit, and difficulty understanding other people. He did not report any depressive symptoms (Table 2).

During the individual session, the occupational therapist leader noted that Lennart did most activities together with his wife, except from participating in a board meeting once a month. His goal was to walk to that meeting on his own, since it was close to home. After recommendations from the occupational therapist leader, he used walking sticks to prevent falls, and successfully fulfilled his goal. During the individual phone survey, Lennart expressed that the program had been helpful and increased his participation in activities outside home, and the leaders had motivated him to try out new strategies (Table 5).

Lennart perceived less impact of the stroke on participation before BUS TRIPS than after, according to SIS but it had decreased at follow-up. At baseline, he scored maximum general self-efficacy, which decreased at the following assessments. He was satisfied with life in general throughout, but even more at follow-up (Figure 1).

Carl is living with his wife in a single-family home. He walks slowly and uses a rollator in- and outdoors. He has access to a car but had been advised not to drive. Before the stroke, he travelled by bus about once a month. Carl identified that access to transport and the design of the environment affected his societal participation. Carl scored below normal cognition, but did not report any such impairments subjectively. He had no depressive symptoms (Table 2).

Although Carl stated that he was interested in travelling by bus before BIS TRIPS, during the program he seemed to change his mind and did not try to travel. He said: “I don’t think I have the need of it” (Carl). He expressed it was difficult to change buses, and due to prior experiences he did not have confidence in the drivers. During the program, the occupational therapist leader noted that Carl needed repeated information of its purpose, and that due to lack of insight and motivation he should not have been admitted. Although Carl did not practice travelling by bus during BUS TRIPS, he expressed that the program had motivated him to try out new strategies and helped him to come closer to travel by bus (Table 5). For example, afterwards Carl expressed no fear to take the bus: “I don’t feel scared about it” (Carl) and that his wife could help him with the bus card. He had also discovered advantages with travelling by bus: “We only have 100 m to the station and then the entire Skåne region within reach” (Carl). 

Pre-program, Carl expressed that his stroke affected his participation in society, mostly during activities together with others due to SIS. At discharge, this impact had increased, but decreased again at follow-up. His general self-efficacy increased from baseline to after program completion, but decreased below baseline at follow-up. Throughout, Carl felt quite satisfied with life in general at baseline, which increased to satisfied after the program and at the follow-up (Figure 1). 

Kent is living with his wife in an apartment. He is a very slow walker and uses mobility devices in- and outdoors. He does not have access to a car. He seldom travelled by bus prior to the stroke. Kent said access to transportation and the environment affected his participation in society. Subjectively, he reported faster tiredness, loss of concentration, and attention and memory deficits. He also reported some depressive symptoms (Table 2). 

Kent was determined to travel by bus again, as pre-stroke when he had managed on his own: “I will have that as a goal, to go out again and take the bus down town” (Kent). According to the occupational therapist leader that traveled with him, Kent was concerned about his self-efficacy when travelling. His goal was to walk from home to the bus stop, to travel to the city center. Kent managed the practice trip himself, according to his plans. Afterwards, he formulated a new goal: to take the trip independently to get a haircut. Kent was satisfied with the support during the program, and expressed it had meant a lot to him and made him feel more secure: “It (the program) has given me incredibly much. I feel much stronger now” (Kent). He expressed how the leader who had traveled with him (occupational therapist) was skilled, and had a personality that made him feel secure: “She feels so knowledgeable and sends out an aura of security which I love and need” (Kent). It was important for him to feel self-confident to travel again—an ability he now mastered: “It’s about overcoming the fear, that I don’t have to be afraid” (Kent). The program had made him travel more by bus by himself and participate more in activities outside home, due to the individual phone survey (Table 5). He felt he had adapted strategies during the program such as splitting up goals into targets, and learnt problem-solving skills. 

Kent perceived the stroke had affected his societal participation at all three data collections, especially during active leisure activities according to SIS. His general self-efficacy was higher at discharge and follow-up than at baseline. He was very satisfied with life in general throughout, except from a small decrease after the program completion (Figure 1).

## 4. Discussion

This study examined an intervention targeting travelling by bus for people with CIs after stroke, an area where there is a lack of research. Our study shows that the new intervention BUS TRIPS is feasible, and the five participants improved their ability to travel by bus, although this could not be conclusively verified by the assessments. Moreover, as highlighted by the results, there are challenges in relation to participant recruitment to consider. 

The participants appreciated the program, and especially valued the group format, confirming previous studies showing benefits of social support in such programs [16]. They also prized the use of a simple and understandable language confirming former studies when recruiting people with stroke in interventions [43]. Practice in real world environments with support from skilled leaders was particularly important, which has also been emphasized in previous research [44]. The participants ranked the program material highly, which indicate that they actually assimilated the program content and used their newfound skills. The positive opinions about the homework, where the participants felt welland encouraged to practice new skills in their everyday environment, confirms SM principles and speaks to the overall feasibility of BUS TRIPS.

Two of the participants (Elisabeth and Kent) were especially motivated to travel, able to formulate clear realistic goals, and ready for the action stage, including the whole travel chain. Since the same two persons who objectively (according to MoCa) did not qualify for moderate or severe CIs, it is worth considering if BUS TRIPS in its current form is best suited for those with no moderate or severe CIs. Some of the other participants did not travel by bus at all during BUS TRIPS. However, some made other progresses; for example, one saw benefits of travelling by bus and another reached a goal of walking independently to a meeting, which in the long run is a skill required to catch a bus. It is not always the tasks related to the bus as such that are perceived as challenging, but other links in the travel chain such as walking to the bus stops. Considering the stages of Prochaska’s and DiClemente’s change model [45], our results show that all participants had developed their readiness to change in relation to travel by bus to some extent. The group format also includes the benefits of social support and peer learning [16], which in turn can improve readiness to change. It is also important to allow enough time during sessions for discussions within the group, which is another reason for lengthening each session. 

Although no statistical conclusions can be drawn from our small sample, a limitation of this study is that, unlike the results of the semi-structured group interview and the individual phone survey data, the assessments (SIS, GSE, and LiSat-11, Item 1) could not conclusively verify any improvements post-program. A noteworthy finding is that four of the five participants scored lower on the SIS [36] after program completion. Possibly, an immediate reaction was that they became aware of their limited ability to move around in society. The use of SIS as an outcome measure should still be considered in larger forthcoming effect studies of the BUS TRIPS, but the choice of outcome measures need to be carefully considered.

The finding that general self-efficacy in our sample was slightly lower than in a general stroke sample [46] and did not indicate any general positive trend throughout the evaluation period deserves a comment. It is possible that BUS TRIPS does not affect general self-efficacy, although no firm conclusions can be drawn based on only five participants. Bandura [47] argued that self-efficacy is related to a specific task and not a general phenomenon. Therefore, it is worth considering whether a self-efficacy assessment directed to travelling by bus, outdoor mobility, and participation could be more responsive to change. To our knowledge, no such instrument exists. Overall, it is a challenge to find suitable outcome measures in rehabilitation research [48] with potential to capture effects following self-management interventions. 

Focusing on meaningful activities is important for people with strokes’ ability to adapt to their new situation [49]. We used the COPM [27] during the first session, to identify problems and goals in relation to performance of travelling by bus activities meaningful for each participant. Although the participants received support during this assessment, there was too little time to administer the COPM during the group session. Instead, participants need to meet with an experienced occupational therapist who can administer this assessment pre-program with each participant. That kind of individual support can facilitate goal setting on meaningful activities, which could potentially improve the outcome of BUS TRIPS. Furthermore, the fact that participants needed more time could be related to their CIs. One lesson learned is that it is important to have two leaders during each session to make time for more individual support. 

All participants in this study lived together with a spouse, but we did not ask them about what role their spouse played in their attempts to travel by bus. This might be a limitation of this study, since research has shown both encouraging and restrictive influences of family and friends when it comes to outdoor mobility and travelling [12,15]. It is also important to consider that, even if many spouses and family members want to be involved in health care interventions, being a caregiver is often also a burden and stress [50]. 

Other study limitations include challenges regarding the recruitment of participants leaving us with a very small sample. Since measurements of restrictions in cognition and reintegration in societal participation are not included in the national stroke register in Sweden, such information was not available during the recruitment to our study. Thus, we could not direct our recruitment to only people with CIs and difficulties related to travelling by bus, but our recruitment had to be from a broader spectrum. On the other hand, previous stroke research has shown that recruiting participants with narrow inclusion criteria is a difficult task [51], and often very few potential participants are identified anyway. One participant withdrew and did not show up for the program due to a lack of energy, which is also a reason for not travelling with public transport [8] and for refraining from participation since post-stroke fatigue is common [52]. Moreover, despite the fact that SM programs offer social support [16], it is possible that people with stroke with CIs hesitate to attend group rehabilitation due to an inclination to expose their CIs to others. 

Further challenges relate to those who actually attended the program. Some of the participants were not motivated to fulfill the tasks in the program, and little insight as to their CIs was another potential barrier for participation. These aspects influenced the program but reflected the complexity when addressing people with stroke with various types of impairments. Recruitment issues are not unique to this study, and people with stroke can be particularly vulnerable and difficult to recruit [51]. There is a need for more research to investigate how people with stroke reason in relation to attending evaluation studies of rehabilitation programs. There is also a need to distinguish potential participants who have come further in their readiness to change from those who are not motivated or have no insight regarding their impairments. 

One strength of our study is the mixed methods approach including different perspectives (i.e., participant, leader), types of data, and analyses. This gave detailed information as to program feasibility and as to how the program might contribute to an improved readiness to travel by bus in the target group. This knowledge will be used to improve BUS TRIPS in future studies.

## 5. Conclusions

The BUS TRIPS program is feasible and has potential to contribute to a readiness to travel by bus for people with stroke and CIs. All participants in our pilot study made some progress, e.g., overcame the fear of travelling by bus or saw advantages some travelling by bus, although not all assessments captured the improvements. Future studies should focus on recruitment challenges, larger scale clinical trials and subsequent implementation in clinical practice.

## Figures and Tables

**Table 1:** Overview of the BUS TRIPS intervention: targeted skills, content, and homework.

	Targeted Skill	Content of Session	Homework
**Session 1**	Self-monitoring	Introduction, cognitive disabilities after stroke, and consequences for daily life—outdoor mobility, bus travels in urban areas	Individual identification of activities problematic for outdoor mobility and bus travels
**Session 2**	Goal-setting	Reviewing homework Goal plan and goal-setting	Setting goals for the intervention period
**Session 3**	Problem solving/Decision making	Presentation of Problem Solving Theory (PST) Teaching activity analysis and compensatory solutions (such as travel planner and mobile phones)	Use goals in rehabilitation plan—find solutions, use PST
**Sessions 4–5**	Problem solving/Decision making	Individually with OT or PT—practice bus travelling in real life context, use goal plan, practice technical devices walking techniques, etc.	Use goals—practice strategies from individual session with OT/PT ^1^ in other activities
**Session 6**	Communication	Share experiences from the individual sessions, Discuss communication with others	Practice communicating problems and asking for help
**Session 7**	Positive thinking	Conclusion, set long-term objectives	

^1^ OT/PT (occupational therapist/physiotherapist).

**Table 2:** Overview of participant profiles, *N* = 5.

Name	Elisabeth	Viola	Lennart	Carl	Kent
Age (year)	64	77	71	82	67
Stroke type	Hemorrhage	Infarction	Infarction	Infarction	Infarction
Living situation	Living with partner	Living with partner	Living with partner	Living with partner	Living with partner
Type of housing	Single-family home	Apartment building	Single-family home	Single-family home	Apartment building
Living area	Urban	Urban	Sub-rural	Sub-rural	Urban
Mobility device					
Indoor	No	No	No	Rollator	Cane or Rollator
Outdoor	Rollator	Rollator	No	Rollator	Sticks or Rollator
Medical recommendation not to drive due to stroke	No	No	No	Yes	No
Access to car in the household	Yes, but do not drive herself	No	Yes, but do not drive himself	Yes, but do not drive himself	No
Bus ride frequency prior stroke	Daily	Several times a week	Rarely	Once a month	Rarely
TUG ^1^	16	18	12	13	24
CHIEF, *m* (range, 0–8) ^2^					
Attitudes/Support	0	0.8	0	0	0.2
Service/Assistance	0	0.6	0	0.4	0.9
Physical/Structural	1.5	2	0	0.3	2.3
Work/School	0	0	0	0	0
Policies	0	0	0	0.5	0
Self-reported cognitive functional limitations ^3^ (range, 0–20)	3	9	3	0	9
MoCa (range, 0–30) ^4^	Normal cognition (28)	Cognitive impairment (18)	Cognitive impairment (18)	Cognitive impairment (18)	Normal cognition (26)
GDS (range, 0–20) ^5^	Possible depression (13)	Possible depression (7)	No depression (2)	No depression (1)	Possible depression (6)
SIS, *m* (range, 1–5) ^6^	2.6	3.8	4.8	3.8	3.3
GSE (range, 10–40) ^7^	28	29	40	31	26
Lisat-11 Item 1 (1–6) ^8^	4	4	5	4	6

^1^ TUG (Timed up and go) [28]: Task usually completed by healthy older people in ten seconds or less. Norm values by age: 60–69 years: 8.1, 70–79 years: 9.2, 80–99 years: 11.3 [29]; ^2^ CHIEF (The Craig Hospital Inventory of Environmental Factors) [30,31]: Higher score = greater frequency and/or magnitude of environmental barriers. A value of 1 or below indicates a small barrier to activity and participation [32]; ^3^ Self-rated cognitive limitations questionnaire, scored 0–20. Higher scores = more cognitive impairments [33]; ^4^ MoCa (Cognitive impairment Montreal cognitive assessment): Normal cognition ≥ 26 [34]; ^5^ GDS (Geriatric depression scale) > 5p = possible depression [35]; ^6^ SIS (Stroke impact scale). Lower scores = more stroke impact on participation [36]; ^7^ GSE (General Self-efficacy scale). Higher scores = higher general self-efficacy [37,38]; ^8^ Lisat-11 (Life satisfaction scale), Item 1. Higher scores = more satisfied [39].

**Table 3:** Data collection sources.

Aim	Feasibility of BUS TRIPS		Improved Ability to Travel by Bus Due to BUS TRIPS	
Analysis	Cross-case		Within-case (Narratives)	
Sample	Participants (*n* = 5)	Leaders (*n* = 2)	Participants (*n* = 5)	Leaders (*n* = 2)
Method (X)	Quantitative	Qualitative	Quantitative	Qualitative
(1) Demographics			X	
(2) Baseline data				
Activity and participation				
Study specific questions on walking devices, transfer possibilities, bus travels			X	
TUG ^1^			X	
Environmental barriers				
CHIEF ^2^			X	
Cognitive impairments				
MoCa ^3^			X	
Self-reported cognitive functional limitations ^4^			X	
Depressive symptoms				
GDS ^5^			X	
(3) Assessments				
SIS ^6^			X	
GSE ^7^			X	
LiSat-11 (Item 1) ^8^			X	
(4) Leaders reflection notes, group sessions		X		
(5) Leaders reflection notes, individual session				X
(6) Semi-structured group-interview		X		X
(7) Individual phone survey	X		X	

^1^ TUG (Timed Up and Go Test) [28]; ^2^ CHIEF (Craig Hospital Inventory of Environmental Factors) [30,31]; ^3^ MoCa (Montreal Cognitive Assessment) [34]; ^4^ Self-rated cognitive functional limitations questionnaire [33]; ^5^ GDS (Geriatric depression scale) [35]; ^6^ SIS (Stroke impact scale) [36]; ^7^ GSE (General Self-Efficacy scale) [37,38]; ^8^ Lisat-11 (Life Satisfaction Questionnaire-11), Item 1 [39].

**Table 4:** Main category and sub-categories of program feasibility.

Main Category	Sub-Category
Program delivery	-Appreciated group format despite too short sessions
	-Importance of skilled leaders and motivated participants
Program content	-Session material adequate but needs minor revision to fit target group
	-Homework is valuable but reflective group discussions must be supported

**Table 5:** The phone survey of the five participants including questions on a seventh grade scale (1 = Not at all and 7 = Excellent).

Survey Questions	Median (Min–Max)
Were the leaders knowledgeable in the subject?	6 (5–7)
Could the leaders present the course material in a way you understood?	6 (5–7)
Did the leaders manage you to try out new strategies?	5 (4–6)
How was the quality of the course material?	5 (4–6)
How was the quality of the homework?	5 (5–7)
How did you experienced the feedback you received at the homework?	4 (4–6)
Where the leaders able to support discussions among the participants?	4 (3–6)
How did you experience the format of the program?	6 (5–6)
Overall, how much do you think this program have helped you to travel more by bus?	5 (4–7)
Overall, how much do you think this program have helped you become more involved in activities outside your home?	5 (4–6)

## References

[1] Adamit T., Maeir A., Ben Assayag E., Bornstein N.M., Korczyn A.D., Katz N. (2015). Impact of first-ever mild stroke on participation at 3 and 6 month post-event: The TABASCO study. Disabil. Rehabil..

[2] Combs S.A., Van Puymbroeck M., Altenburger P.A., Miller K.K., Dierks T.A., Schmid A.A. (2013). Is walking faster or walking farther more important to persons with chronic stroke?. Disabil. Rehabil..

[3] Balakrishnan R., Kaplan B., Negron R., Fei K., Goldfinger J.Z., Horowitz C.R. (2017). Life after stroke in an urban minority population: A photovoice project. Int. J. Environ. Res. Public Health.

[4] Broome K., McKenna K., Fleming J., Worrall L. (2009). Bus use and older people: A literature review applying the Person-Environment-Occupation model in macro practice. Scand. J. Occup. Ther..

[5] Haak M., Fänge A., Hortsmann V., Iwarsson S. (2008). Two dimensions of participation in very old age and their relations to home and neighborhood environments. Am. J. Occup. Ther..

[6] Tan K.M., O’Driscoll A., O’Neill D. (2011). Factors affecting return to driving post-stroke. Ir. J. Med. Sci..

[7] Asplund K., Wallin S., Jonsson F. (2012). Use of public transport by stroke survivors with persistent disability. Scand. J. Disabil. Res..

[8] Wendel K., Ståhl A., Risberg J., Pessah-Rasmussen H., Iwarsson S. (2010). Post-stroke functional limitations and changes in use of mode of transport. Scand. J. Occup. Ther..

[9] Risser R., Lexell E.M., Bell D., Iwarsson S., Ståhl A. (2015). Use of local public transport among people with cognitive impairments—A literature review. Transp. Res. Part F Traffic Psychol. Behav..

[10] Wretstrand A., Ståhl A. (2008). User Needs and Expectations Relative to Accessible Transport: Framework for Mobility Planning.

[11] Ståhl A., Månsson Lexell E. (2017). Facilitators for traveling with local public transport among people with mild cognitive limitations after stroke. Scand. J. Occup. Ther..

[12] Logan P.A., Armstrong S., Avery T.J., Barer D., Barton G.R., Darby J., Gladman J.R., Horne J., Leach S., Lincoln N.B. (2014). Rehabilitation aimed at improving outdoor mobility for people after stroke: A multicentre randomised controlled study (the Getting out of the House Study). Health Technol. Assess..

[13] White J.H., Miller B., Magin P., Attia J., Sturm J., Pollack M. (2012). Access and participation in the community: A prospective qualitative study of driving post stroke. Disabil. Rehabil..

[14] Rosenkvist J., Risser R., Iwarsson S., Ståhl A. (2010). Exploring mobility in public environments among people with cognitive functional limitations—Challenges and implications for planning. Mobilities.

[15] Barnsley L., McCluskey A., Middleton S. (2012). What people say about travelling outdoors after their stroke: A qualitative study. Aust. Occup. Ther. J..

[16] Lorig K.R., Holman H. (2003). Self-management education: History, definition, outcomes, and mechanisms. Ann. Behav. Med..

[17] Jones F., Riazi A., Norris M. (2013). Self-management after stroke: Time for some more questions?. Disabil. Rehabil..

[18] Huijbregts M.P., Myers A.M., Streiner D., Teasell R. (2008). Implementation, process, and preliminary outcome evaluation of two community programs for persons with stroke and their care partners. Top. Stroke. Rehabil..

[19] Kendall E., Catalano T., Kuipers P., Posner N., Buys N., Charker J. (2007). Recovery following stroke: The role of self-management education. Soc. Sci. Med..

[20] Warner G., Packer T., Villeneuve M., Audulv A., Versnel J. (2015). A systematic review of the effectiveness of stroke self-management programs for improving function and participation outcomes: Self-management programs for stroke survivors. Disabil. Rehabil..

[21] Wolf T.J., Baum C.M., Lee D., Hammel J. (2016). The development of the improving participation after stroke self-management program (IPASS): An exploratory randomized clinical study. Top. Stroke Rehabil..

[22] Jones F., Riazi A. (2011). Self-efficacy and self-management after stroke: A systematic review. Desabil. Rehabil..

[23] Shevil E., Finlayson M. (2009). Process evaluation of a self-management cognitive program for persons with multiple sclerosis. Patient Educ. Couns..

[24] Shevil E. (2008). Developing and Pilot Testing a Cognitive Intervention Program for Persons with Multiple Sclerosis. Ph.D. Thesis.

[25] Lindén A., Lexell J., Larsson Lund M. (2011). Improvements of task performance in daily life after acquired brain injury using commonly available everyday technology. Disabil. Rehabil. Assist. Technol..

[26] Craig P., Dieppe P., Macintyre S., Michie S., Nazareth I., Petticrew M. (2006). Developing and Evaluating Complex Interventions: New Guidance. http://www.mrc.ac.uk/documents/pdf/complex-interventions-guidance/.

[27] Law M., Polatajko H., Pollock N., McColl M.A., Carswell A., Baptiste S. (2006). Canadian Occupational Performance Measure.

[28] Podsiadlo D., Richardson S. (1991). The timed “Up & Go”: A test of basic functional mobility for frail elderly persons. J. Am. Geriatr. Soc..

[29] Bohannon R.W. (2006). Reference values for the timed up and go test: A descriptive meta-analysis. J. Geriatr. Phys. Ther..

[30] Lund M.L., Lexell J. (2009). Associations between perceptions of environmental barriers and participation in persons with late effects of polio. Scand. J. Occup. Ther..

[31] Whiteneck G.G., Harrison-Felix C.L., Mellick D.C., Brooks C.A., Charlifue S.B., Gerhart K.A. (2004). Quantifying environmental factors: A measure of physical, attitudinal, service, productivity, and policy barriers. Arch. Phys. Med. Rehabil..

[32] Zhang L., Yan Y., You L., Li K. (2015). Barriers to activity and participation for stroke survivors in rural China. Arch. Phys. Med. Rehabil..

[33] Wendel K., Risberg J., Pessah-Rasmussen H., Ståhl A., Iwarsson S. (2008). Long-term cognitive functional limitations post stroke: Objective assessment compared with self-evaluations and spouse reports. Int. J. Rehabil. Res..

[34] Nasreddine Z.S., Phillips N.A., Bédirian V., Charbonneau S., Whitehead V., Collin I., Cummings J.L., Cherkow H. (2005). The Montreal Cognitive Assessment, MoCA: A brief screening tool for mild cognitive impairment. J. Am. Geriatr. Soc..

[35] Gottfries G.G., Noltorp S., Nørgaard N. (1997). Experience with a Swedish version of the Geriatric Depression Scale in primary care centres. Int. J. Geriatr. Psychiatry.

[36] Duncan P.W., Wallace D., Lai S.M., Johnson D., Embertson S., Laster L.J. (1999). The stroke impact scale version 2.0. Evaluation of reliability, validity, and sensitivity to change. Stroke.

[37] (1999). Swedish Version of the General Self-Efficacy Scale. http://userpage.fu-berlin.de/~health/swedish.htm.

[38] Schwarzer R., Jerusalem M., Weiman J., Wright S., Johnston M. (1995). Generalized self-efficacy scale. Measures in Health Psychology: A User’s Portfolio. Causal and Control Beliefs.

[39] Fugl-Meyer A.R., Melin R., Fugl-Meyer K.S. (2002). Life satisfaction in 18- to 64-year-old Swedes: In relation to gender, age, partner and immigrant status. J. Rehabil. Med..

[40] Yin R.K. (2014). Case Study Research: Design and Methods.

[41] Creswell J.W., Plano Clark V.L. (2011). Designing and Conducting Mixed Methods Research.

[42] Hsieh H.F., Shannon S.E. (2005). Three approaches to qualitative content analysis. Qual. Health Res..

[43] Hadidi N., Buckwalter K., Lindquist R., Rangen C. (2012). Lessons learned in recruitment and retention of stroke survivors. J. Neurosci. Nurs..

[44] Patterson F., Fleming J., Doig E. (2016). Group-based delivery of interventions in traumatic brain injury rehabilitation: A scoping review. Disabil. Rehabil..

[45] Prochaska J.O., DiClemente C.C., Norcross J.C., Goldfried M.R. (2005). The Transtheoretical approach. Handbook of Psychotherapy Integration.

[46] Carlstedt E., Lexell E.M., Pessah-Rasmussen H., Iwarsson S. (2015). Psychometric properties of the Swedish version of the General Self-Efficacy Scale in stroke survivors. Int. J. Rehabil. Res..

[47] Bandura A. (1997). Self-Efficacy: The Exercise of Control.

[48] Lejeune T.M., Stoquart G.G. (2015). The challenge of assessment in rehabilitation. J. Rehabil. Med..

[49] Woodman P., Riazi A., Pereira C., Jones F. (2014). Social participation post stroke: A meta-ethnographic review of the experiences and views of community-dwelling stroke survivors. Disabil. Rehabil..

[50] Bakas T., Clark P.C., Kelly-Hayes M., King R.B., Lutz B.J., Miller E.L. (2014). Evidence for stroke family caregiver and dyad interventions: A statement for healthcare professionals from the American Heart Association and American Stroke Association. Stroke.

[51] Boxall L., Hemsley A., White N. (2016). Exploring recruitment issues in stroke research: A qualitative study of nurse researchers’ experiences. Nurse Res..

[52] Choi-Kwon S., Kim J.S. (2011). Poststroke fatigue: An emerging, critical issue in stroke medicine. Int. J. Stroke.

